# Nonlinear fourth-order elastic characterization of the cornea using torsional wave elastography

**DOI:** 10.1007/s13246-023-01314-8

**Published:** 2023-08-29

**Authors:** Antonio Callejas, Inas Faris, Jorge Torres, Guillermo Rus

**Affiliations:** 1 Ultrasonics Lab (TEP-959), Department of Structural Mechanics, University of Granada, Granada, 18071 Spain; 2 TEC-12 group, Instituto de Investigación Biosanitaria, ibs.Granada, 18001 Spain; 3 Excellence Research Unit “ModelingNature” (MNat), Universidad de Granada, Granada, 18001 Spain

**Keywords:** Fourth-order elastic properties, Cornea, Torsional wave elastography (TWE), Intraocular pressure (IOP), Nonlinearity

## Abstract

Measuring the mechanical nonlinear properties of the cornea remains challenging due to the lack of consensus in the methodology and in the models that effectively predict its behaviour. This study proposed developing a procedure to reconstruct nonlinear fourth-order elastic properties of the cornea based on a mathematical model derived from the theory of Hamilton et al. and using the torsional wave elastography (TWE) technique. In order to validate its diagnostic capability of simulated pathological conditions, two different groups were studied, non-treated cornea samples (n=7), and ammonium hydroxide ($NH_4OH$) treated samples (n=7). All the samples were measured in-plane by a torsional wave device by increasing IOP from 5 to 25 mmHg with 5 mmHg steps. The results show a nonlinear variation of the shear wave speed with the IOP, with higher values for higher IOPs. Moreover, the shear wave speed values of the control group were higher than those of the treated group. The study also revealed significant differences between the control and treated groups for the Lamé parameter $\mu$ (25.9–6.52 kPa), third-order elastic constant *A* (215.09–44.85 kPa), and fourth-order elastic constant *D* (523.5–129.63 kPa), with p-values of 0.010, 0.024, and 0.032, respectively. These findings demonstrate that the proposed procedure can distinguish between healthy and damaged corneas, making it a promising technique for detecting diseases associated with IOP alteration, such as corneal burns, glaucoma, or ocular hypertension.

## Introduction

Maintenance of external ocular morphology and function is related to the biomechanical properties of the cornea [1]. Several corneal disorders, such as keratoconus, ectatic disorders, and chemical eye burns, emerge as the most important due to their severity, impact on patient quality of life, and incidence [2–4]. Alkali burns are the most common emergency related to ocular trauma [5–7] (84$\%$ of cases [4]), resulting in high visual morbidity [8]. Significant corneal damage was determined when the pH was greater than 11.5 [9]. Among the most common alkali chemical agents, ammonium hydroxide ($NH_4OH$) has the fastest penetration rate (<3 min) [10]. As a consequence, chemical injuries of the eye produce extensive damage to the ocular surface epithelium, cornea, anterior segment, and limbal stem cells resulting in permanent unilateral or bilateral visual impairment [11]. Damage to the corneal and conjunctival epithelium may lead to opacification and neo-vascularization of the cornea [11], causing the appearance of an acute increase in IOP due to shrinkage and contraction.

To provide clinically relevant critical information for the diagnosis of corneal diseases, elastography is proposed as an emerging non-invasive imaging method. Very recently, Optical Coherence Elastography (OCE) was used to detect the propagation of induced elastic waves to biomechanically assess the cornea [12–16], lens [17] and retina [18]. The advantages of this and other recent elastography techniques include the microscale sensitivity in motion detection [19–21] and the noncontact approach, along with the image’s microscale resolution. However, the direction of vibration of the particles, nearly perpendicular to the median plane of the cornea, results in the generation of guided waves motivated by the relationship between wavelength and tissue thickness, which prompts the use of complex guided wave models [22]. As additional disadvantages are the low frame rate in 2D imaging, long acquisition imaging times, and exact positioning where slight motion could cause image artifacts [23, 24].

Beyond the standard of elasticity maps, a more precise, pressure- and operator-independent interpretation of the measurements might be obtained considering nonlinearity since the dependence of the stiffness modulus with deformation is correlated with IOP pressure. In this sense, nonlinear elastic constants may be much more sensitive to specific diseases, facilitating an early diagnosis [25]. In addition, most biological tissues have a nonlinear stress–strain behavior under variable amounts of pressure [26]. This nonlinear elasticity can cause biological tissues to become more stressed under increased pressure [27].

The formula proposed by Hamilton et al. [28], a strain energy equation that neglects compression terms, has been applied to the mechanical characterization of soft tissues both with the acoustoelasticity technique [29] and in uniaxial tensile tests [27, 30] to determine nonlinear elastic parameters. In acoustoelasticity, Hamilton’s formula was used for the breast [26], liver [31, 32], or porcine kidney [33] considering only the shear modulus ($\mu$) and the third-order elastic constant (*A*), and neglecting the fourth-order elastic constant (*D*) because it was hypothesized that the shear wave displacements were smaller than static compression. According to cornea applications, the influence of the pressure applied in the tissue in determining its stiffness has been studied. In this regard, ex-vivo studies have demonstrated that the stiffness of the cornea and sclera increases with increasing the intraocular pressure [34–38]. Regarding the nonlinear characterization of the cornea, to our knowledge, few studies have been performed. An ex-vivo study has been carried out to characterize nonlinear parameters of the cornea using uniaxial destructive tests [27]. However, to date, no work has studied Hamilton-Zabolotskaya’s fourth-order parameters in the cornea employing non-destructive tests, with potential application in clinical practice.

Torsional waves are postulated to be a crucial tool, sensitive to the measurements of nonlinear parameters [25, 39]. Based on the studies presented, there is a knowledge gap about the effectiveness of the different nonlinear analysis methods that can be applied to the existing TWE technique. This work proposes a mathematical model based on Hamilton et al. theory [28] for the porcine cornea nonlinear behavior characterization of two groups, non-treated cornea samples and samples treated with an ammonium hydroxide concentration ($NH_4OH$), using the TWE technique. The geometry of the wave emitting part and the receiving part are adapted to the curvature of the cornea, an evolution from the original design used in previous studies for the characterization of cervical tissue [40, 41]. An important focus is the vibration of the particles due to torsion waves. In this case, said vibration is contained in the median plane of the cornea (in-plane propagation). A distinctive feature of this technique was that we obtained information about in-plane shear deformation in the cornea, therefore, the influence of guided waves, likely present in out-of-plane propagation disappears [34], along with the associated complexity, unlike the OCE technique (out-of-plane propagation).

The remainder of this paper is organized as follows. Section 2 describes the preparation of samples, the Torsional Wave Elastography technique, the proposed mathematical model, and the data post-processing. Sections 3 and 4 describe the results and the discussion, respectively. Finally, Section 5 offers the conclusions of this research and suggestions for the future.

## Materials and methods

### Tissue preparation

Due to the similarity of the mechanical behavior between porcine samples and the human ones [42], in this study, porcine eye globes were used to characterize the nonlinear behavior of the cornea. Fourteen porcine eye globes were enucleated postmortem from a local abattoir and placed immediately in phosphate-buffered saline solution (PBS, pH 7.4) to maintain tissue hydration and to prevent the cornea from hardening until Torsional Wave Elastography measurements were performed. The solution was prepared using di-Sodium Hydrogen Phosphate anhydrous (Reag. Ph. Eur. 99%), Potassium di-Hydrogen Phosphate (Reag. Ph. Eur. 99% purity), and Sodium Chloride (USP, BP, Ph. Eur. JP 99%) from Panreac AppliChem. All samples were tested at room temperature within eight hours postmortem. Exposures to household cleaning products commonly involve the eyes. This exposure occurred in 8.4% according to a study carried out in United States [43]. Ammonium hydroxide ($NH_4OH$) solution (EMSURE ACS, Reag. Ph Eur 28-30%) is one of the typical cleaning products. For that reason, two different groups were studied, non-treated cornea samples (n=7) and $NH_4OH$-treated samples (n=7). Ammonium hydroxide was mixed with distilled water in a similar concentration (3 mM $NH_4OH$ at 10% v/v) to that of cleaning products [44]. The exposure time for the treated samples was 5 min, while no treatment was applied to the control group. After that, all the treated samples were washed in PBS before examination with TWE.

### Torsional wave elastography

The same torsional wave device was used as in the study carried out by Torres et al. [34], where the sensor (receiving ring - 4 mm base) and excitation (emitting disk - external and internal diameters were 13 mm and 9.6 mm respectively) components were assembled. Torsional waves were generated by rotating the emitter in contact with the cornea, which are a type of shear waves that propagate axisymmetrically until they reach the receiver. The displacement peaked at around 2 $\mu$m. The geometry of the sensor was optimized so that the volumetric displacement components were negligible compared to the shear displacement components, and this is achieved using a receiving ring whose center coincides with the center of the emitting disk [45, 46]. That configuration makes it possible to obtain, after the analysis of the received signal, the shear mechanical parameters in-plane that interrogate the properties of the cornea. In addition, the receiving ring had an internal curvature that was adapted to the geometry of the cornea [47].

**Fig. 1:**
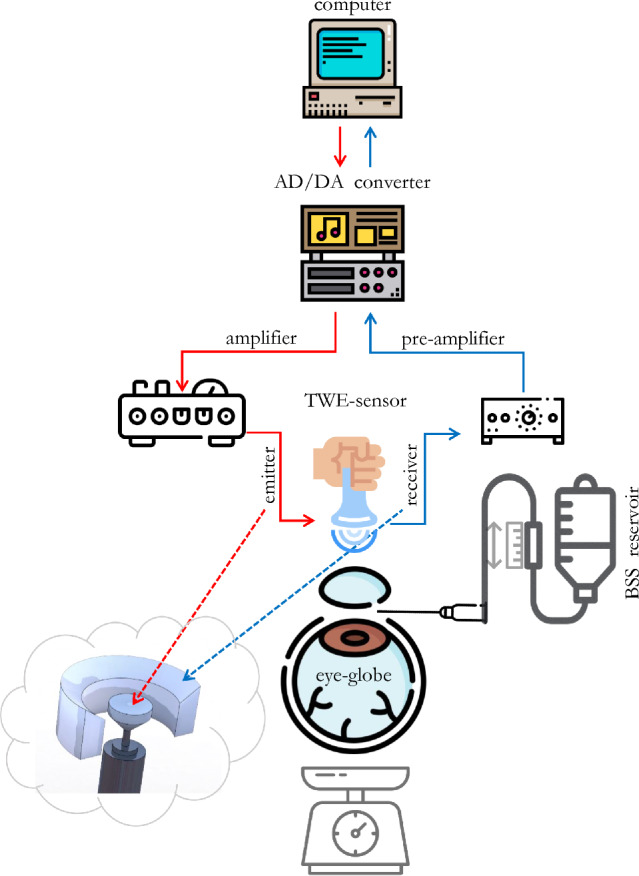
Experimental setup for the nonlinear mechanical characterization of the cornea using TWE

The diagram of the experimental configuration is shown in Fig. 1. A multichannel AD/DA converter with a 192 kHz sampling rate was employed to emit the signal and receive it after the propagation through the cornea. All the samples were measured with a single sinusoidal pulse of 1000 Hz, according to previous references in the literature [40, 48]. An amplifier was used to output a 25V peak-to-peak signal. The received signal by the TWE probe was preamplified (40 dB gain) before reaching the digital to analog converter. In order to reduce random and high-frequency noise, an average of 16 signals and a 5 kHz low-pass filter were applied. The total measurement time was 3.2 s, composed of 16 intervals of 200 ms. A calibration measurement was then subtracted from the received cornea signal to counterbalance crosstalk effects. All elements were computer-controlled using high-speed communication ports and a Matlab environment (R2018b, The MathWorks Inc., Natick, MA, USA). Based on previous results, no guided waves were produced in-plane shear deformation caused by TWE measurement [34]. Therefore, the phase speed was calculated by subtracting a quarter of the period (inverse of the received signal central frequency) from the first signal peak. Due to the small propagation distance between the emitter and the receiver (2.8 mm), the curvature of the cornea was approximated as a straight line [49].

Each eyeball was placed in a custom-made holder and the axis of the emitter was aligned with the optical axis of the eyeball. A needle was inserted into the anterior chamber of the eye, which was connected to a saline solution reservoir. The IOP was modulated by adjusting the height of the reservoir [50]. The tests increased IOP from 5 to 25 mmHg with steps of 5 mmHg. The IOP was held constant at each IOP level for 2 min before TWE measurements. All corneas were measured three times by repositioning the probe. The pressure applied with the TWE probe was small enough so that the received wave had the higher possible amplitude to avoid slippery conditions [41] and did not influence the IOP controlled by the saline solution reservoir.

### Mathematical model

**Fig. 2:**
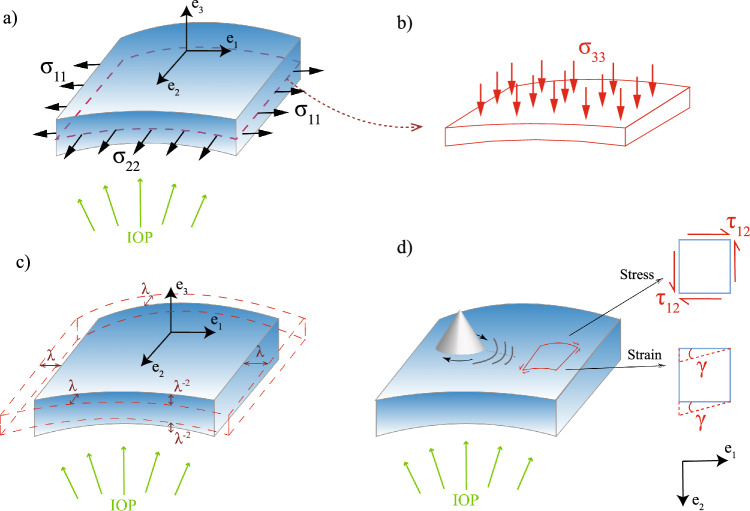
Scheme of the stress and strain field. A differential element of the cornea is represented in blue. Subfigures a and b show the normal stresses to which the cornea is subjected in three directions according to the reference system adopted ($e_1, e_2, e_3$). The strain field is representated in Subfigure c. Finally, subfigure d shows the in-plane strain generated by the torsion wave

Considering the deformation due to the intraocular pressure and that due to shear wave propagation (see Fig. 2), the deformation gradient tensor is given by:

1 $$\begin{aligned} F= \begin{bmatrix} \lambda &{} \gamma &{} 0 \\ \gamma &{} \lambda &{} 0 \\ 0 &{} 0 &{} \lambda ^{-2} \end{bmatrix} \end{aligned}$$

where $\lambda$ is the stretch (defined as $\lambda =1+\epsilon$, $\epsilon$ is the strain) in the two circumferential directions and a strain of $\lambda ^{-2}$ in the radial direction, which ensures incompressibility ($J=det(F)=1$). In addition, torsional waves generate shear strain in the cornea, which is given by $\gamma (x_{12},t)$ ($x_{12}$ shear strain direction - Fig. 2, *t* time), considering that the strain due to the torsional wave is much smaller than the strain induced by changing the IOP (i.e., $\gamma<< \epsilon$).

The Green-Lagrange strain tensor is given by:

2 $$\begin{aligned} E=\dfrac{1}{2}(F^{T}\cdot F - I)=\dfrac{1}{2} \begin{bmatrix} (\lambda ^{2}-1) &{} 2\gamma \lambda &{} 0 \\ 2\gamma \lambda &{} (\lambda ^{2}-1) &{} 0 \\ 0 &{} 0 &{} (\lambda ^{-4}-1) \end{bmatrix} \end{aligned}$$

The strain energy density equation proposed by Hamilton et al. [28] is,

3 $$\begin{aligned} W=\mu I_2 + \frac{1}{3} A I_3 + D I_2^2 \end{aligned}$$

Where $I_2$ and $I_3$ are the invariants of the strain tensor, $\mu$ is the Lamé constant, *A* is the third-order elastic constant, and *D* is the fourth-order elastic constant. These terms are sufficient for describing shear deformation when the energy stored in compression is comparatively insignificant. The invariants are calculated as,

4 $$\begin{aligned} I_2= & {} tr({\textbf {E}}^2) \end{aligned}$$

5 $$\begin{aligned} I_3= & {} tr({\textbf {E}}^3) \end{aligned}$$

The second Piola-Kirchhoff (PK) stress tensor may be computed as $S=\partial W/\partial E$. Using this equation, equation 3, and taking into account that $\gamma<< \varepsilon$ [34, 51] we obtain (see Appendix (Sect. 6) for more details),

6 $$\begin{aligned}{} & {} S_{ij}=\mu \dfrac{\partial I_{2}}{E_{ij}} +\dfrac{1}{3}\,A\,\dfrac{\partial I_{3}}{E_{ij}} + D\,\dfrac{\partial I_{2}^{2}}{E_{ij}} \end{aligned}$$

7 $$\begin{aligned}{} & {} S_{11}=\mu (\lambda ^{2}-1) +\dfrac{A}{4}(\lambda ^{2}-1)^{2}\nonumber \\{} & {} \qquad +D\left[ (\lambda ^{2}-1)^{3} +(\lambda ^{2}-1)\dfrac{(\lambda ^{-4}-1)^{2}}{2}\right] \end{aligned}$$

Considering the porcine cornea as a spherical shell of radius ($R = 8.45 mm$) [47], and a typical thickness of a porcine cornea ($\tau = 1 mm$), the equilibrium equation is,

8 $$\begin{aligned} \sigma _{11}=\frac{IOP \cdot R}{2 \cdot \tau } \end{aligned}$$

where *IOP* is the intraocular pressure in mm of Hg, and $\sigma$ is the Cauchy stress tensor.

The relation between the Cauchy stress tensor and the second Piola-Kirchhoff stress tensor is,

9 $$\begin{aligned}{} & {} S=J\cdot F^{-1} \,\cdot \sigma \cdot F^{-T} \end{aligned}$$

10 $$\begin{aligned}{} & {} \quad S_{11}\nonumber \\{} & {} \quad =\dfrac{\sigma _{11}}{\lambda ^{2}}= \boxed { \dfrac{IOP\cdot R}{2 \,\tau \,\lambda ^{2}}=\mu (\lambda ^{2}-1) +\dfrac{A}{4}(\lambda ^{2}-1)^{2} +D\left[ (\lambda ^{2}-1)^{3}+(\lambda ^{2}-1)\dfrac{(\lambda ^{-4}-1)^{2}}{2}\right] } \end{aligned}$$

To obtain a relationship between the torsional wave speed ($c_{s}$) and the stretch, the Green-Lagrange strain tensor has been decomposed into static ($E^{s}$) and dynamic ($E^{d}$) strain as follows,

11 $$\begin{aligned} E=E^{s} + E^{d} = \begin{bmatrix} \dfrac{(\lambda ^{2}-1)}{2} &{} 0 &{} 0 \\ 0 &{} \dfrac{(\lambda ^{2}-1)}{2} &{} 0 \\ 0 &{} 0 &{} \dfrac{(\lambda ^{-4}-1)}{2} \end{bmatrix} + \begin{bmatrix} \dfrac{1}{2}u_{2,1}^{2} &{} \dfrac{1}{2} u_{2,1} &{} 0 \\ \dfrac{1}{2}u_{2,1} &{} 0 &{} 0 \\ 0 &{} 0 &{} 0 \end{bmatrix} \end{aligned}$$

The dynamic strain has been obtained considering the displacements associated with an in-plane ($e_{1}e_{2}$) torsional wavefront.

12 $$\begin{aligned} E_{ij}^d=\dfrac{1}{2}\left( \dfrac{\partial u_{i}}{\partial x_{j}}+\dfrac{\partial u_{j}}{\partial x_{i}}+\displaystyle \sum _{k}\dfrac{\partial u_{k}}{\partial x_{i}}\cdot \dfrac{\partial u_{k}}{\partial x_{j}} \right) \end{aligned}$$

Taking into account the following shear wave equation,

13 $$\begin{aligned} \rho \, \ddot{u}_{2} = S_{12,1} \end{aligned}$$

We arrive at a nonlinear relationship between the shear wave speed ($c_s$) and the parameters $\mu$, *A*,and *D*:

14 $$\begin{aligned} \boxed { c_{s}^{2} \cdot \rho = \mu \, + \dfrac{A}{2}(\lambda ^{2}-1)+D\left[ (\lambda ^{2}-1)^{2}+\dfrac{(\lambda ^{-4}-1)^{2}}{2}\right] } \end{aligned}$$

For a given value of *IOP* and the $\mu$, *A*, and *D* parameters, we can obtain $\lambda$ using equation 10. Afterwards, the corresponding torsional wave speed ($c_s$) and the value of $\lambda$ are introduced to minimize the equation 14 and obtain the parameters $\mu$, *A* and *D*. It is iterated until the error of the three aforementioned parameters is minimized for each sample of each group with genetic algorithms (Matlab - R2018b, The MathWorks Inc., Natick, MA, USA).

## Results

**Fig. 3:**
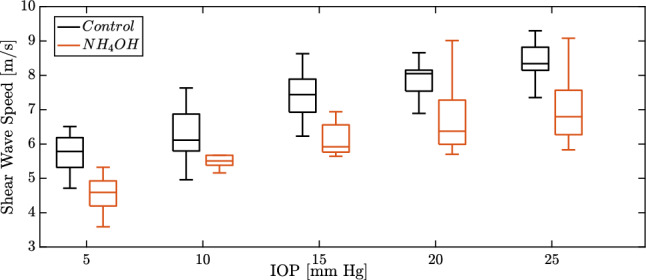
Comparison of the shear wave speed (in m/s) from TWE (Torsional Wave Elastography) for both groups (control - black solid line boxplots and $NH_4OH$ - red solid line boxplots), and intraocular pressure (IOP in mmHg)

Figure 3 shows the shear wave speed of the TWE technique for both groups, control and $NH_4OH$, and for each IOP (5-25 mmHg with 5 mmHg steps). We observed that the values of the control group were higher than those of the treated group. Furthermore, the shear wave speed varied nonlinearly with the IOP, with an increase in the speed observed as the IOP increased.

**Fig. 4:**
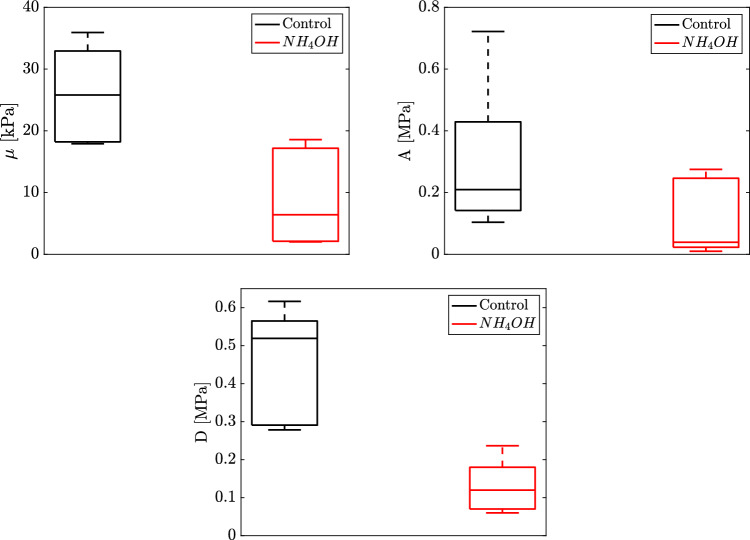
Reconstructed values from the mathematical model proposed (Eqs. 10, and 14). Up to the left) Lamé parameter ($\mu$) $p=0.010$. Up to the right) Third-order elastic constant (*A*) $p=0.024$. Bottom) Fourth-order elastic constant (*D*) $p=0.032$. The values of the three reconstructed parameters were higher in the control group (black solid line boxplots) than in the treated ($NH_4OH$) group (red solid line boxplots). The difference between the three reconstructed parameters for the two groups was significant

The parameters reconstructed from the proposed model (Eqs. 10, and 14) for the control and $NH_4OH$ groups are displayed in Fig. 4. The boxplots of the Lamé parameter $\mu$, third-order elastic constant *A*, and fourth-order elastic constant *D* showed higher values in the control group. The Mann–Whitney-Wilcoxon test was used to study the significance level of the reconstructed parameters for both groups. A value of $p<0.05$ was considered statistically significant for the difference testing. We observed that the difference between the Lamé parameter $\mu$, third-order elastic constant *A*, and fourth-order elastic constant *D* for the two groups was significant, $p=0.010$, $p=0.024$, and $p=0.032$ respectively.

**Fig. 5:**
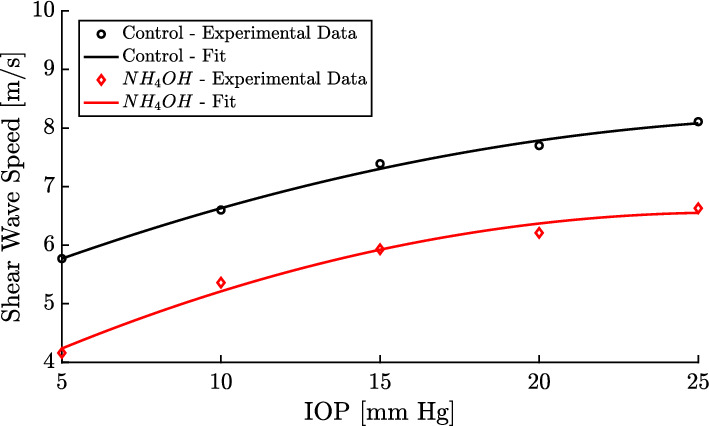
Fit of experimental data with the proposed mathematical model. Control sample ($\mu =25.54$ kPa, $A=767$ kPa, $D=303$ kPa) - $R^2=0.995$. Treated sample with $NH_4OH$ ($\mu =7.33$ kPa, $A=238$ kPa, $D=152$ kPa) - $R^2=0.983$

Fig. 5 displays the representative fit (comparing shear wave speed to IOP) of the experimental data for both a control sample and an $NH_4OH$-treated sample using our proposed mathematical model. The reconstructed parameters for the control fit were $\mu =25.54$ kPa, $A=767$ kPa, $D=303$ kPa, while for the $NH_4OH$ fit were $\mu =7.33$ kPa, $A=238$ kPa, $D=152$ kPa. Across all samples from both groups, the coefficient of determination ($R^2$) demonstrated a strong fit with a minimum value of 0.9.

**Fig. 6:**
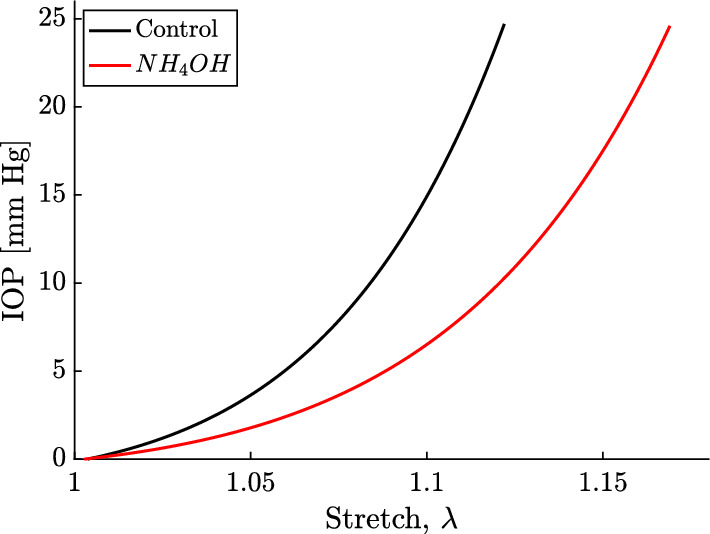
Relationship between stretch and IOP. Black solid line: control ($\mu =25.54$ kPa, $A=767$ kPa, $D=303$ kPa). Red solid line: treated with $NH_4OH$ ($\mu =7.33$ kPa, $A=238$ kPa, $D=152$ kPa)

The relationship between stretch and IOP was represented for control and $NH_4OH$ cornea samples using the Eq. 10 for a given $\mu$, *A*, and *D* parameters (see Fig. 6). It is clear that the IOP varies nonlinearly with the stretch and that the slope of the curve, which is determined by the nonlinear parameters *A* and *D*, is higher in the control group.

## Discussion

Several studies have suggested that nonlinearity could enhance the current diagnosis in elastography since the operator dependency is decoupled by correlating the stiffness and the tissue deformation [25]. In this study, a mathematical model based on Hamilton et al. theory [28] is proposed for the porcine cornea nonlinear behavior characterization of two groups, seven non-treated cornea samples and seven samples treated with $NH_{4}OH$, using the TWE technique.

Recent work has focused on corneal mechanical characterization. Weng et al. [52] used a high-frequency ultrasound elastography system based on an external vibrator and ultrafast ultrasound imaging to estimate the viscoelasticity of porcine cornea ex-vivo by using a Lamb wave model. A study carried out by Torres et al. [34] developed an elastography technique based on torsional waves (TWE) adapted to the specificities of the cornea to characterize the viscoelasticity of two groups, a control group and one group of alkali burn treatment ($NH_{4}OH$). The TWE technique reflected mechanical properties changes after treatment, showing a high potential for clinical diagnosis due to its rapid performance time.

According to the nonlinear characterization of the cornea, few studies have been performed. By using a similar experimental setup than [52], Zhang J. et al. [53] assessed the nonlinear elastic properties of the cornea and ciliary body using the hyperelastic Blatz model. However, they did not compare the nonlinear response of the healthy with other model corneas. Ashofteh Y. et al. [27] evaluated which hyperelastic model (Hamilton-Zabolotskaya model, Ogden model, and Mooney-Rivlin model) could best describe the nonlinear behavior of the cornea to discriminate structural changes in a damaged cornea (*NaOH* treatment) with quasi-static uniaxial tensile tests. However, they did not study the nonlinearity of the damaged cornea with one of the most common alkali chemical agents and the fastest penetration rate (ammonium hydroxide - $NH_{4}OH$). Another disadvantage is that the uniaxial tensile test is a destructive procedure, unlike our study. Thus, the cornea samples were characterized by high deformation ratios.

The correlation between the shear wave speed and the (IOP) was studied for both groups, the control, and $NH_4OH$ group, and for each IOP 5-25 mmHg with 5 mmHg steps, see Fig. 3. Shear wave speed values for the control group are higher than those of the treated group. This was also evidenced in the work carried out by Torres et al. [34], which is motivated by significant changes in the composition of the extracellular matrix of the cornea after a corneal chemical injury [54]. Collagen fibrils behave nonlinearly for high intraocular pressure values [55]. Results from Fig. 3 show how the shear wave speed depends on the IOP nonlinearly, which is consistent with previous literature studies [34, 55].

A complex microstructure governs the nonlinear behavior of the cornea. It is composed of a hydrated proteoglycan matrix with collagen fibrils embedded in it [56]. The collagen fibrils are packed and arranged in the circumferential direction [57, 58]. The TWE technique used in this work interrogates the mechanical properties of the cornea in the plane e1-e2 (in-plane - deformations produced by the torsion wave in that plane, see Fig. 2), which correspond with the collagen fibrils of the cornea, unlike the OCE and ultrasound techniques which measured in the plane e1-e3 or e2-e3 (out-of-plane), the matrix of the cornea. Given this, our technique’s advantage lies in the wave propagation, not generating Lamb waves, which was demonstrated in the work carried out by Torres et al. [34]. However, Lamb waves are generated by measuring out of the plane, and a more complex study is required [52, 59]. Figure 3 shows how the reconstructed values of shear wave speed for the untreated cornea are higher than previous studies present in the literature [52, 53, 53] for different values of IOP, which may be motivated by in-plane (plane e1-e2) measurements.

In recent years, there has been a growing interest in the mechanical characterization of the cornea. Researchers, such as Weng et al., have employed a high-frequency ultrasound elastography system to estimate the viscoelasticity of the porcine cornea ex-vivo. Similarly, Torres et al. developed an elastography technique based on torsional waves that was adapted to the specificities of the cornea. The TWE technique demonstrated its potential for clinical diagnosis due to its rapid performance time and ability to reflect mechanical property changes after treatment. While some studies have focused on the nonlinear characterization of the cornea, such as Zhang J. et al.’s assessment of the nonlinear elastic properties of the cornea and ciliary body using the hyperelastic Blatz model, few have compared the nonlinear response of healthy corneas to other model corneas. Ashofteh Y. et al. evaluated which hyperelastic model could best describe the nonlinear behavior of the cornea to discriminate structural changes in a damaged cornea, yet they did not study the nonlinearity of the damaged cornea with one of the most common alkali chemical agents and the fastest penetration rate. Furthermore, the uniaxial tensile test is a destructive procedure, which sets our study apart as the cornea samples were characterized by high deformation ratios. Our study proposes a nonlinear fourth-order elastic model that can differentiate between healthy and damaged corneas. We found significant differences in elastic constants between the control and NH4OH groups. The TWE technique has the potential to measure nonlinear parameters and detect diseases related to intraocular pressure, making it a promising concept with diagnostic potential for diseases like glaucoma. The correlation between the shear wave speed and the intraocular pressure was studied for both groups, the control, and NH4OH group. Shear wave speed values for the control group are higher than those of the treated group. This was also evidenced in the work carried out by Torres et al., who were motivated by significant changes in the composition of the extracellular matrix of the cornea after a corneal chemical injury. Results show how the shear wave speed depends on the intraocular pressure nonlinearly, which is consistent with previous literature studies. The nonlinear behavior of the cornea is governed by a complex microstructure. In this study, the TWE technique interrogated the mechanical properties of the cornea in the plane e1-e2, which corresponded with the collagen fibrils of the cornea. This is in contrast to other techniques such as OCE and ultrasound, which measured in the matrix of the cornea. Our technique’s advantage lies in the wave propagation, which reconstructs values of shear wave speed for the untreated cornea that are higher than previous studies present in the literature for different values of intraocular pressure. This may be motivated by in-plane measurements.

The aforementioned nonlinear fourth-order elastic model is based on the strain energy density equation by Hamilton et al. [28], which allows a nonlinear fourth-order elastic characterization of the cornea using Torsional Wave Elastography. The model is governed by the Lamé constant ($\mu$), the third-order elastic constant (*A*), and the fourth-order elastic constant (*D*). These terms are sufficient for describing shear deformation when the energy stored in compression is comparatively insignificant. This is achieved because torsional waves present the advantage of isolating a pure shear motion, thus, eliminating the generation of spurious compressional waves due to the configuration emitter-receiver of the probe [45, 60]. Genetic algorithms (Matlab - R2018b, The MathWorks Inc., Natick, MA, USA) were employed to minimize the discrepancy between the pairwise measurements of torsional wave speed ($c_s$) and *IOP*, equations described in detail in the Mathematical model section (Eqs. 10, and 14). Figure 4 shows the boxplots of the values of the Lamé parameter $\mu$, third-order elastic constant *A*, and fourth-order elastic constant *D* for both groups, control, and $NH_4OH$ group.

The reconstructed parameters for the control group fit were $\mu =25.9 \pm 6.85$ kPa, $A=215.09 \pm 133.58$ kPa, $D=523.50 \pm 161.35$ kPa, while for the $NH_4OH$ group fit $\mu =6.52 \pm 3.94$ kPa, $A=44.85 \pm 13.30$ kPa, $D=129.63 \pm 76.09$ kPa. A minimum value of 0.9 for the coefficient of determination ($R^2$) was obtained for both groups’ samples. Generally, the reconstructed parameters for the control group are notably higher than those for the $NH_4OH$ group. This discrepancy is particularly evident in the parameters *A* and *D* when observed on the Stretch-IOP curve (Fig. 6). The curve shows a steeper slope as the strain in the control corneas increases, providing superior contrast when calculating *A* due to the smaller data deviation compared to the fourth-order elastic parameter *D*. Alireza et al. [27] employed the same nonlinear model, using measurements from uniaxial tensile tests for a control group and a *NaOH*-treated group of porcine corneas. However, the parameters $\mu$, *A*, and *D* values that we obtained for the control group differ from those in their study. This variation could be due to the differences in the stretch analyzed; the strain in their study is higher, and it is known that the shear modulus ($\mu$) increases with the applied strain [55]. However, measurements by Bernal et al. [26] of the nonlinear parameter *A* in tissues align with the values obtained in our study, as the order of magnitude of the shear deformation is similar. To compare the two groups in our study, we applied the Mann–Whitney-Wilcoxon test to assess the significance level of the reconstructed parameters. The differences between the Lamé parameter $\mu$, the third-order elastic constant *A*, and the fourth-order elastic constant *D* for the two groups were significant (p = 0.010, p = 0.024, and p = 0.032, respectively). The threshold for statistical significance was set at $p<0.05$. Despite studies in the literature often disregarding the fourth-order parameter *D* for small deformations [26, 29], our results highlight the potential of parameter *D* as a valuable biomarker for distinguishing between healthy and alkali-burned porcine eyes within the studied deformation range. Figure 6 demonstrates the relationship between the stretch and IOP for both groups, using equation 10 after reconstructing the three nonlinear parameters. This figure depicts the nonlinear relationship between IOP and stretch and shows that the slope of the curve, determined by the nonlinear parameters*A* and *D*, is steeper in the control group.

In Fig. 6, the relationship between the stretch and the IOP was represented for both groups, using equation 10 after reconstructing the three nonlinear parameters. We can observe the nonlinear behavior between IOP and the stretch, and that the slope of the curve, which is determined by the nonlinear parameters *A* and *D*, is higher in the control group.

The proposed method presents some limitations that need to be exposed. The TWE technique only receives a signal; therefore, no 2D image is obtained that could provide us with additional information, such as focal changes in tissue consistency. Besides, it is difficult to change the IOP in conventional examination procedures. However, according to daily IOP fluctuations of the human corneas [61], this technique could be an emerging concept with the diagnostic potential to measure the nonlinear parameters and to detect diseases associated with intraocular pressure such as corneal burns, glaucoma, or ocular hypertension. Ocular inspection requires that the patient does not make involuntary movements that cancel the measurement, which is why the proposed technique has the advantage of measuring in a short period of time. The tonometry technique, currently used in the diagnosis of glaucoma, consists of flattening the cornea to calculate IOP, which can be uncomfortable for the patient [62]. However, the TWE technique only requires a low contact pressure between the probe and cornea to perform the measurement. In addition, such a technique could diagnose diseases related to IOP by correlating it with shear modulus. In future work, the study of the correlation between IOP and shear stiffness for diseases such as glaucoma is proposed using the nonlinear method.

## Conclusions

In our study, we propose a mathematical model utilizing the torsional wave elastography (TWE) technique to characterize the cornea’s nonlinear fourth-order elastic properties based on the theory developed by Hamilton et al. [28]. We conducted our analysis on two groups of cornea samples, one untreated (n=7) and the other treated with ammonium hydroxide ($NH_4OH$) (n=7). Using a torsional wave device to measure in-plane, we incrementally increased the intraocular pressure (IOP) from 5 to 25 mmHg in 5 mmHg increments, and observed that the untreated group had higher shear wave speed values when compared to the treated group. Furthermore, the shear wave speed exhibited nonlinear variation with IOP, with an increase observed as intraocular pressure increased. Our analysis of reconstructed parameters revealed significant differences between the untreated and treated group for the Lamé parameter $\mu$, third-order elastic constant *A*, and fourth-order elastic constant *D*. Additionally, we demonstrated that the stretch exhibited nonlinear variation with IOP. Through the correlation of stiffness and tissue deformation, this technique has the potential to detect diseases associated with IOP alteration, such as corneal burns, glaucoma, or ocular hypertension, by decoupling operator dependency and measuring nonlinear parameters.

## Data Availability

Not applicable.

## Appendix: Mathematical model

The second Piola-Kirchhoff (PK) stress tensor may be computed as ${\textbf {S}}=\partial W/\partial {\textbf {E}}$.

The strain energy density equation proposed by Hamilton et al. [28] is,

15 $$\begin{aligned} W=\mu I_2 + \frac{1}{3} A I_3 + D I_2^2 \end{aligned}$$

where $I_2$ and $I_3$ are the invariants of the strain tensor, $\mu$ is the Lamé constant, *A* the third-order elastic constant, and *D* the fourth-order elastic constant. The invariants are calculated as,

16 $$\begin{aligned} I_2= & {} tr({\textbf {E}}^2) \end{aligned}$$

17 $$\begin{aligned} I_3= & {} tr({\textbf {E}}^3) \end{aligned}$$

Therefore,

18 $$\begin{aligned} S_{11} = \mu \frac{\partial I_2}{\partial \varepsilon _{11}} + \frac{1}{3} A \frac{\partial I_3}{\partial \varepsilon _{11}} +D \frac{\partial I_2^2}{\partial \varepsilon _{11}} \end{aligned}$$

Assuming incompressibility, the deformation gradient tensor is given by:

19 $$\begin{aligned} F= \begin{bmatrix} \lambda &{} \gamma &{} 0 \\ \gamma &{} \lambda &{} 0 \\ 0 &{} 0 &{} \lambda ^{-2} \end{bmatrix} \end{aligned}$$

where $\lambda$ is the stretch (defined as $\lambda =1+\epsilon$, $\epsilon$ is the strain) in the two circumferential directions and a strain of $\lambda ^{-2}$ in the radial direction, which ensures incompressibility ($J=det(F)=1$). In addition, torsional waves generate shear strain in the cornea, which is given by $\gamma (x_{12},t)$ ($x_{12}$ shear strain direction - Fig. 2, *t* time), considering that the strain due to the torsional wave is much smaller than the strain induced by changing the IOP (i.e., $\gamma<< \epsilon$).

The Green-Lagrange strain tensor is given by:

20 $$\begin{aligned} E= & {} \dfrac{1}{2}(F^{T}\cdot F - I)=\dfrac{1}{2} \begin{bmatrix} (\lambda ^{2}-1) &{} 2\gamma \lambda &{} 0 \\ 2\gamma \lambda &{} (\lambda ^{2}-1) &{} 0 \\ 0 &{} 0 &{} (\lambda ^{-4}-1) \end{bmatrix} \end{aligned}$$

21 $$\begin{aligned} E= & {} \dfrac{1}{2} \begin{bmatrix} (\lambda ^{2}-1) &{} 2\gamma \lambda &{} 0 \\ 2\gamma \lambda &{} (\lambda ^{2}-1) &{} 0 \\ 0 &{} 0 &{} (\lambda ^{-4}-1) \end{bmatrix} \end{aligned}$$

The invariants and their respective derivatives to obtain $S_{11}$ are:

22 $$\begin{aligned}{} & {} I_{2}=tr(E^{2})=E_{11}^{2}+E_{12}\cdot E_{21} + E_{12}\cdot E_{21}+ E_{22}^{2} +E_{33}^{2} \end{aligned}$$

23 $$\begin{aligned}{} & {} \quad \dfrac{\partial I_{2}}{\partial E_{11}}=2E_{11}=2\,\dfrac{(\lambda ^{2}-1)}{2}=\lambda ^{2}-1 \end{aligned}$$

24 $$\begin{aligned}{} & {} \quad \dfrac{\partial I_{3}}{\partial E_{11}} =3\,E_{11}^{2} + E_{12}\cdot E_{21} \nonumber \\{} & {} \qquad + E_{12}\cdot E_{21} + E_{21}\cdot E_{12} \simeq 3\,E_{11}^{2} \Rightarrow (\gamma<< \varepsilon ) \end{aligned}$$

25 $$\begin{aligned}{} & {} \quad \dfrac{\partial I_{3}}{\partial E_{11}}\nonumber \\{} & {} \quad =3\,\left( \dfrac{\lambda ^{2}-1}{2}\right) ^{2}=\dfrac{3}{4}(\lambda ^{2}-1)^{2} \end{aligned}$$

26 $$\begin{aligned}{} & {} \begin{aligned} \dfrac{\partial I_{2}^{2}}{\partial E_{11}}&\simeq 4\,E_{11}^{3} + 4\,E_{11}\cdot E_{22}^{2} + 4\,E_{11}\cdot E_{33}^{2} \\&=4\,\left( \dfrac{\lambda ^{2}-1}{2}\right) ^{2} +4\,\left( \dfrac{\lambda ^{2}-1}{2}\right) \left( \dfrac{\lambda ^{2}-1}{2}\right) ^{2}\\&\qquad +4\,\left( \dfrac{\lambda ^{2}-1}{2}\right) \left( \dfrac{\lambda ^{-4}-1}{2}\right) ^{2} \end{aligned} \end{aligned}$$

Therefore,

27 $$\begin{aligned}{} & {} S_{11}=\mu (\lambda ^{2}-1) +\dfrac{A}{4}(\lambda ^{2}-1)^{2}\nonumber \\&\qquad&+D\left[ (\lambda ^{2}-1)^{3}+(\lambda ^{2}-1)\dfrac{(\lambda ^{-4}-1)^{2}}{2}\right] \end{aligned}$$

Considering the porcine cornea as a spherical shell of radius ($R = 8.45 mm$) [47], and a typical thickness of a porcine cornea ($\tau = 1 mm$), the equilibrium equation is,

28 $$\begin{aligned} \sigma _{11}=\frac{IOP \cdot R}{2 \cdot \tau } \end{aligned}$$

where *IOP* is the intraocular pressure in mm of Hg, and $\sigma$ is the Cauchy stress tensor.

The relation between the Cauchy stress tensor and the second Piola-Kirchhoff stress tensor is,

29 $$\begin{aligned} S=J\cdot F^{-1} \,\cdot \sigma \cdot F^{-T} \end{aligned}$$

We obtain a relationship between the intraocular pressure of the cornea and the parameters of the nonlinear model $\mu$, *A*, and *D*.

30 $$\begin{aligned}{} & {} S_{11}\nonumber \\{} & {} \quad =\dfrac{\sigma _{11}}{\lambda ^{2}}= \boxed { \dfrac{IOP\cdot R}{2 \,\tau \,\lambda ^{2}}=\mu (\lambda ^{2}-1) +\dfrac{A}{4}(\lambda ^{2}-1)^{2} +D\left[ (\lambda ^{2}-1)^{3}+(\lambda ^{2}-1)\dfrac{(\lambda ^{-4}-1)^{2}}{2}\right] } \end{aligned}$$

To obtain a relationship between the torsional wave speed ($c_{s}$) and the stretch, the Green-Lagrange strain tensor has been decomposed into static ($E^{s}$) and dynamic ($E^{d}$) strain as follows,

31 $$\begin{aligned}{} & {} E=E^{s} + E^{d} = \begin{bmatrix} \dfrac{(\lambda ^{2}-1)}{2} &{} 0 &{} 0 \\ 0 &{} \dfrac{(\lambda ^{2}-1)}{2} &{} 0 \\ 0 &{} 0 &{} \dfrac{(\lambda ^{-4}-1)}{2} \end{bmatrix}\nonumber \\{} & {} \qquad + \begin{bmatrix} \dfrac{1}{2}u_{2,1}^{2} &{} \dfrac{1}{2} u_{2,1} &{} 0 \\ \dfrac{1}{2}u_{2,1} &{} 0 &{} 0 \\ 0 &{} 0 &{} 0 \end{bmatrix} \end{aligned}$$

The dynamic strain tensor has been obtained considering the displacements associated with an in-plane ($e_{1}e_{2}$) torsional wavefront.

32 $$\begin{aligned} E_{ij}^d=\dfrac{1}{2}\left( \dfrac{\partial u_{i}}{\partial x_{j}}+\dfrac{\partial u_{j}}{\partial x_{i}}+\displaystyle \sum _{k}\dfrac{\partial u_{k}}{\partial x_{i}}\cdot \dfrac{\partial u_{k}}{\partial x_{j}} \right) \end{aligned}$$

Taking into account the following wave equation,

33 $$\begin{aligned} \rho \, \ddot{u}_{2} = S_{12,1} \end{aligned}$$

Next, we calculate the $S_{12,1}$ term of the second Piola-Kirchhoff stress tensor.

34 $$\begin{aligned} S_{12}=\mu \dfrac{\partial I_{2}}{E_{12}} +\dfrac{1}{3}\,A\,\dfrac{\partial I_{3}}{E_{12}} + D\,\dfrac{\partial I_{2}^{2}}{E_{12}} \end{aligned}$$

Now, the invariants and their respective derivatives to obtain $S_{12,1}$:

35 $$\begin{aligned}{} & {} \dfrac{\partial I_{2}}{E_{12}}=\,E_{21}+\,E_{21}=2\,E_{21} \end{aligned}$$

36 $$\begin{aligned}{} & {} \begin{aligned} \dfrac{\partial I_{3}}{\partial E_{12}}&\quad =E_{21}\cdot E_{11} + E_{11}\cdot E_{21} + E_{22}\cdot E_{21}\\&\qquad + E_{11}\cdot E_{21} + E_{22}\cdot E_{21} + E_{22}\cdot E_{21} \\&\quad =3\,E_{11}\cdot E_{21}+3\,E_{22}\cdot E_{21} \end{aligned} \end{aligned}$$

37 $$\begin{aligned}{} & {} \begin{aligned} \dfrac{\partial I_{2}^{2}}{\partial E_{12}}&\quad =E_{11}^{2}\cdot E_{21} + E_{11}^{2}\cdot E_{21}\\&\qquad + E_{11}^{2}\cdot E_{21} +2\, E_{12}\cdot E_{21}^{2} +2\, E_{12}\cdot E_{21}^{2} + E_{21}\cdot E_{22}^{2} \\&\qquad + E_{21}\cdot E_{33}^{2} + E_{21}\cdot E_{11}^{2} +2\, E_{12}\cdot E_{21}^{2}\\&\qquad +2\, E_{12}\cdot E_{21}^{2} + E_{21}\cdot E_{22}^{2} + E_{21}\cdot E_{33}^{2} \\&\qquad + E_{22}^{2}\cdot E_{21} + E_{22}^{2}\cdot E_{21}\\&\qquad + E_{33}^{2}\cdot E_{21} + E_{33}^{2}\cdot E_{21} \\&\quad =4\,E_{11}^{2}\cdot E_{21} + 4\,E_{22}^{2}\cdot E_{21}\\&\qquad + 4\,E_{33}^{2}\cdot E_{21} + 8\,E_{12}\cdot E_{21}^{2} \end{aligned} \end{aligned}$$

38 $$\begin{aligned}{} & {} \begin{aligned} S_{12}&\quad =2 \,\mu E_{12} +\dfrac{1}{3}\,A\,(3\,E_{11}\cdot E_{11} + 3\,E_{22}\cdot E_{21} )\\&\qquad + D\,(4\,E_{11}^{2}\cdot E_{21} + 4\,E_{22}^{2}\cdot E_{21}\\&\qquad + 4\,E_{33}^{2}\cdot E_{21} + 8\,E_{12}\cdot E_{21}^{2}) \end{aligned} \end{aligned}$$

The relationship between the derivative of the dynamic strain tensor terms and the displacements associated with the shear wave propagation was obtained with the equation 32.

39 $$\begin{aligned} \bigg \vert \begin{aligned}&E_{11,1}^d=\dfrac{1}{2}\,2\, u_{2,1}\cdot u_{2,11}= u_{2,1}\cdot u_{2,11}\\&E_{22,1}^d=E_{33,1}^d=0\\&E_{12,1}^d=\dfrac{1}{2}\,u_{2,11} \end{aligned} \end{aligned}$$

Deriving and simplifying the expression for $S_{12}$,

Substituting the displacements from equation 39,

$$\begin{aligned} \begin{aligned}&S_{12,1}=2 \,\mu \,\dfrac{1}{2}\, u_{2,11} +A\,\left[ u_{2,1}\cdot u_{2,11} \cdot \dfrac{1}{2}u_{2,1} +\dfrac{1}{2} u_{2,11} \left( \dfrac{\lambda ^{2}-1}{2}\right. \right. \\&\qquad \left. \left. +\dfrac{1}{2}u_{2,1}^{2} \right) +\left( \dfrac{\lambda ^{2}-1}{2}\cdot \dfrac{1}{2}u_{2,11} \right) \right] \\&\qquad + D \, \Bigg [ 8\cdot \left( \left( \dfrac{\lambda ^{2}-1}{2}+\dfrac{1}{2}\, u_{2,1}^{2}\right) \cdot \left( u_{2,1}\cdot u_{2,11}\right) \cdot \frac{1}{2} u_{2,1}\right) \\&\qquad + 4\,\left( \dfrac{\lambda ^{2}-1}{2}+\dfrac{1}{2}\, u_{2,1}^{2}\right) ^{2}\cdot \frac{1}{2} u_{2,11} \\&\qquad + 4\,\left( \dfrac{\lambda ^{2}-1}{2}\right) ^{2}\cdot \frac{1}{2} u_{2,11} + 4\,\left( \dfrac{\lambda ^{-4}-1}{2}\right) ^{2}\cdot \frac{1}{2} u_{2,11}\\&\qquad +8\cdot \dfrac{1}{2}\, u_{2,11}\cdot \left( \dfrac{1}{2}\, u_{2,1}\right) ^{2}\\&\qquad +16 \left( \dfrac{1}{2}\, u_{2,1}\right) ^{2}\cdot \frac{1}{2} u_{2,11} \Bigg ] \end{aligned} \end{aligned}$$

neglecting the infinitesimal terms,

$$\begin{aligned} \begin{aligned}&S_{12,1}=\mu \, u_{2,11} +A\,\left[ \dfrac{1}{4}\cdot (\lambda ^{2}-1)\, u_{2,11} +\dfrac{1}{4} \cdot (\lambda ^{2}-1)\, u_{2,11} \right] \\&\qquad + D \, \left[ \dfrac{4}{2}\cdot \left( \dfrac{\lambda ^{2}-1}{2} \right) ^{2} \,u_{2,11}\right. \\&\qquad \left. +\dfrac{4}{2}\cdot \left( \dfrac{\lambda ^{2}-1}{2} \right) ^{2} \,u_{2,11}\right. \\&\qquad \left. +\dfrac{4}{2}\cdot \left( \dfrac{\lambda ^{-4}-1}{2} \right) ^{2} \,u_{2,11} \right] \end{aligned} \end{aligned}$$

After considering equation 33, we arrive at a nonlinear relationship between the shear wave speed ($c_s$) and the parameters $\mu$, *A*,and *D*:

$$\begin{aligned} c_{s}^{2}= & {} \dfrac{\mu \, + A \left[ \dfrac{(\lambda ^{2}-1)}{2}\right] +D\left[ 4\left( \dfrac{\lambda ^{2}-1}{2}\right) ^{2} +2\left( \dfrac{\lambda ^{-4}-1}{2}\right) ^{2}\right] }{\rho } \\{} & {} \boxed { c_{s}^{2} \,\rho =\mu \, + A \left[ \dfrac{(\lambda ^{2}-1)}{2}\right] +D \left[ (\lambda ^{2}-1)^{2}+\dfrac{(\lambda ^{-4}-1)^{2}}{2}\right] } \end{aligned}$$
